# Validity evidence of the Hospital Consumer Assessment of Healthcare Providers and Systems instrument: Brazilian version

**DOI:** 10.1590/0034-7167-2024-0526

**Published:** 2026-08-03

**Authors:** Gislene Aparecida Xavier Reis, Laura Misue Matsuda, Flavio Rebustini, Diovane Ghignatti da Costa, Maria do Carmo Fernandez Lourenço Haddad, Sonia Silva Marcon

**Affiliations:** I Universidade Estadual de Maringá. Maringá, Paraná, Brazil; II Universidade de São Paulo. São Paulo, São Paulo, Brazil; III Universidade Federal de Santa Catarina. Florianópolis, Santa Catarina, Brazil; IV Universidade Estadual de Londrina. Londrina, Paraná, Brazil

**Keywords:** Patient-Centered Care, Patient Participation, Outcome Assessment, Health Care, Validation Study, Psychometrics., Atención Dirigida al Paciente, Participación del Paciente, Evaluación de Resultado en la Atención de Salud, Estudio de Validación, Psicometría.

## Abstract

**Objectives:**

to adapt and validate the Hospital Consumer Assessment of Healthcare Providers and Systems instrument for Brazilian culture.

**Methods:**

a methodological study involving translation, cross-cultural adaptation, content validity, response process, and evaluation of the internal structure of the instrument across six dimensions. Data were collected remotely by sending a link via WhatsApp to access the adapted instrument.

**Results:**

during the cross-cultural adaptation, some terms required adjustment; in the content validity phase, certain items were excluded and grammatical modifications were made. No changes were indicated in the response process. In the exploratory factor analysis, 13 items (59%) were excluded, and evidence suggested structural issues in the instrument, indicating it was composed of a single dimension. The confirmatory factor analysis confirmed that the instrument is unidimensional and lacks precision.

**Conclusions:**

the use of this adapted version for Brazilian culture is not recommended.

## RESUMEN

**Objetivos:**

adaptar y validar el instrumento *Hospital Consumer Assessment of Healthcare Providers and Systems* para la cultura brasileña.

**Métodos:**

estudio metodológico que incluyó traducción, adaptación transcultural, validez de contenido, proceso de respuesta y evaluación de la estructura interna del instrumento en seis dimensiones. Los datos fueron recolectados de forma remota mediante el envío de un enlace por WhatsApp para acceder al instrumento adaptado.

**Resultados:**

durante la adaptación transcultural, fue necesario ajustar algunos términos; en la fase de validez de contenido, se excluyeron ciertos ítems y se realizaron modificaciones gramaticales. No se indicaron cambios en el proceso de respuesta. En el análisis factorial exploratorio, se excluyeron 13 ítems (59%) y se observó evidencia de problemas estructurales en el instrumento, lo que indicó que estaba compuesto por una única dimensión. El análisis factorial confirmatorio ratificó que el instrumento es unidimensional y carece de precisión.

**Conclusiones:**

no se recomienda el uso de esta versión adaptada para la cultura brasileña.

## INTRODUCTION

With the advent of widespread information dissemination regarding the healthcare system, patients have become increasingly aware of their rights. As a result, it is essential for services in this field to implement initiatives focused on patient quality and safety^(1,2)^.

Among the strategies for assessing the quality of care, those in which patients report their experiences with the assistance received stand out^(2,3)^. Specifically, patient experience (PE) refers to the outcome of their interactions with the healthcare service and the organizational culture throughout their care journey^(4)^.

Another way to evaluate care is through instruments that specifically address patient experience. One such instrument is the Hospital Consumer Assessment of Healthcare Providers and Systems (HCAHPS^®^), developed and validated in 2008 by the Agency for Healthcare Research and Quality (AHRQ), in partnership with the Centers for Medicare & Medicaid Services (CMS)^(5)^ and Agency For Healthcare Research and Quality^(6)^. In addition to assessing PE, HCAHPS^®^ enables hospital organizations to receive financial subsidies from the U.S. government, allows healthcare consumers to choose the hospital where they wish to receive medical care, facilitates performance comparisons among hospitals, and promotes greater transparency in the quality of care across healthcare services^(5,6)^.

It is timely to validate this instrument for Brazilian Portuguese, not only due to the importance of evaluating PE for quality and safety in care, but also because the results may be useful for hospitals, given that the questionnaire addresses comprehensive aspects of the care received and is self-administered.

This study is guided by the following question: *Does the Hospital Consumer Assessment of Healthcare Providers and Systems instrument, after undergoing cross-cultural adaptation and validation, show evidence of reliability for Brazilian Portuguese*?

## OBJECTIVES

Validate the Hospital Consumer Assessment of Healthcare Providers and Systems instrument for Brazilian Portuguese.

## METHODS

### Ethical Aspects

All ethical principles related to research involving human subjects were respected, and the proposal for this investigation was approved by the ethics committee of the affiliated institution (Approval No. 4.608.997).

### Study Setting and Period

A high-complexity hospital located in the southern region of Brazil, operating as a philanthropic institution, with 311 beds-60% of which are allocated to patients from the *Sistema Único de Saúde* (*“SUS”*, Unified Health System). The hospital provides care in surgical clinic, pediatrics, gynecology and obstetrics, adult, pediatric, and neonatal intensive care units. It holds a Level II quality certification issued by the National Accreditation Organization. Data collection took place between April and October 2021.

### Type of Study

A methodological study involving cross-cultural adaptation^(7)^ and validation of the psychometric properties^(8)^ of the HCAHPS instrument. The original version of the instrument consists of 29 questions distributed across six domains/dimensions: communication with the nursing staff (04); communication with the medical team (03); hospital environment (02); hospital experience (05); discharge-related aspects (06); overall hospital rating (02); sociodemographic characteristics and current health status of the patient (07). This research was structured according to the Revised Standards for Quality Improvement Reporting Excellence (SQUIRE).

### Cross-Cultural Adaptation of *Hospital Consumer Assessment of Healthcare Providers and Systems*

The HCAHPS instrument was translated with authorization from its original authors and underwent cross-cultural adaptation (CCA)^(7)^, following the steps of translation, synthesis of translations, back-translation, evaluation by an Expert Committee, pre-testing of the adapted version, and final validation by the original authors. For the translation phase, two bilingual Brazilian translators carried out independent translations, one specialized in the field and the other a “naïve” translator, with no prior knowledge of the concepts. Both submitted reports on the process. Based on these translations, the researcher, with support from the translators, developed a consensus version.

In the back-translation phase, two native English-speaking translators, unfamiliar with the subject matter, retranslated the instrument and produced reports. The Expert Committee^(9)^, composed of 11 members, including translators, a language professor, and patient safety specialists, was selected through the Lattes Curriculum platform, the *REBRAENSP* website, and LinkedIn, based on academic qualifications (master’s or doctoral degrees) and a minimum of one year of experience in the field. Upon acceptance, participants received the Informed Consent Form and documents related to the adaptation process online, and completed a questionnaire assessing idiomatic, semantic, experiential, and conceptual equivalence.

### Content Validity

All materials produced during the cross-cultural adaptation were submitted for review by 11 experts, including: one translator, one language professor, and nine specialists in patient safety, patient experience, or patient-centered care, each with at least six months of practical or academic experience. The committee did not include psychometricians, whose contribution could have strengthened the technical and statistical analysis of the instrument. This absence occurred because, at the time, the researcher did not yet have full command of the methodological criteria recommended by the Standards for Educational and Psychological Testing^(8)^, which emphasize the importance of including psychometricians in such processes.

Following the qualitative analysis, a quantitative assessment was conducted using the Item-Level Content Validity Index (I-CVI), calculated with the formula: I-CVI = number of responses rated “3” or “4” / total number of responses. A minimum score of 0.80 was considered acceptable, with a preferred threshold above 0.90. The experts’ suggestions were incorporated, resulting in the pre-final version of the HCAHPS, which was approved by the AHRQ team.

### Response Process

The pre-final version of the HCAHPS was sent via WhatsApp to patients who met the criteria established by CMS^(5)^ and AHRQ^(6)^: age ≥ 18 years, clinical, surgical, or obstetric hospitalization, and discharge between 48 hours and six weeks. Patients without WhatsApp contact or who did not respond after at least three contact attempts were not included. Potential participants were identified through daily analysis of electronic medical records, considering diagnosis, WhatsApp contact information, and discharge date. Patients were invited to participate in the study through a written WhatsApp message explaining the study’s purpose, data collection method, and estimated time to complete the questionnaire.

Those who agreed to participate received, via the same channel, a link to the Informed Consent Form, Sociodemographic Questionnaire, Instructions, and the pre-final version of the HCAHPS. After completion, telephone interviews were conducted using a structured questionnaire to assess item comprehension. Data were recorded in an Excel spreadsheet and analyzed using absolute and relative frequency. All participants demonstrated understanding of the instrument’s items, thus concluding the pre-test phase.

### Internal Structure

To assess the internal structure, the final version of the instrument was administered to a new sample of patients who met the same inclusion criteria previously established. The data were compiled in a Microsoft Excel^®^ spreadsheet, exported to the Factor Analysis software (version 10.10.03), and subjected to exploratory factor analysis (EFA), followed by confirmatory factor analysis (CFA).

Initially, Pearson correlation was performed, as the data did not converge in the polychoric correlation matrix due to inconsistencies in the response scales: items with dichotomous responses (10, 12, 16, 17); five-point Likert scale items (4 and 22); items with a “skip” option (11, 13, 14, 16, 17); an item with three categories (15); and an item with 11 categories (18). Additionally, the items with a “skip” option generated 72.7% missing data, increasing the inconsistency of the results. It is worth noting that the initial analysis was only possible after excluding item 10, as the software was unable to process calculations with its inclusion.

The following criteria were used to verify sample adequacy: Kaiser-Meyer-Olkin (KMO) coefficient > 0.70; Bartlett’s test of sphericity with p < 0.05; matrix determinant > 0.00001; and bootstrapping with 5,000 resamples^(10)^.

Parallel Analysis was used in the exploratory factor analysis (EFA) to examine the dimensionality of the HCAHPS. Unweighted Least Squares (ULS) was applied as the extraction method, Promin factor rotation was used to test multidimensionality, and no rotation technique was applied for one-dimensionality^(10)^. Factors were evaluated based on factor loadings ranging from 0.30 to 0.70; communalities between 0.40 and 0.70; and Heywood cases < 0% or > 100%. Regarding the confirmatory factor analysis (CA), values close to 1 were considered acceptable for the Normed Fit Index and Comparative Fit Index; values > 0.90 for the Goodness of Fit Index and Adjusted Goodness of Fit Index; and values close to zero for the Root Mean Square Error of Approximation^(11)^.

Reliability was analyzed using Standardized Cronbach’s Alpha and McDonald’s Omega, with indices ranging from 0.70 to 0.90; Construct Reliability - Index G H (latent and observed), with values > 0.80^(11)^. To confirm the dimensionality of the HCAHPS, the following techniques were applied: Unidimensional Congruence ≥ 0.95, Explained Common Variance ≥ 0.85, and Mean of Item Residual Absolute Loading ≤ 0.30, respectively^(11)^.

Regarding the quality and effectiveness of the model, the following criteria were considered: Factor Determinacy Index > 0.90, Marginal EAP Reliability > 0.80, Sensitivity Ratio > 2, and **Expected Percentage of True Differences > 90%**^(10)^. It is worth noting that this sequence of techniques was guided by the study of Mialhe et al.^(12)^, whose reference values for EFA were based on Hair et al.^(10)^ and Brown^(11)^ for CFA.

## RESULTS

### Cross-Cultural Adaptation

**Figure 1:**
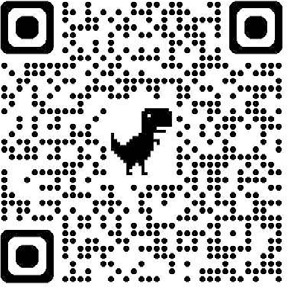
Version of the Hospital Consumer Assessment of Healthcare Providers and Systems produced during the cross-cultural adaptation stage, Maringá, Paraná, Brazil, 2021

During the translation consensus meeting, it was agreed to use the terms “*pressionei*” instead of “*acionei*”; “*próxima*” instead of “*ao redor*”; and “comadre” instead of “*urinol*”. The version of the HCAHPS produced during the cross-cultural adaptation phase is available via the QR code in Figure 1.

### Content Validity

Among the 11 experts/judges, the majority were female (81.8%), nurses (72.7%), with experience in quality, safety, and/or patient experience in the hospital setting (90.9%). More than half held a doctoral degree (54.5%) and resided in the southern region of Brazil (54.5%). In the first round of evaluation, the Expert Committee identified the need for grammatical adjustments in items 2, 11, 16, and 20 to meet the criteria of semantic, experiential, idiomatic, and conceptual equivalence. Additionally, items 23 to 29 were deemed inconsistent and unrelated to the latent variable and were therefore excluded. The Content Validity Index (CVI) obtained was 0.40.

After the proposed adjustments, a second round of evaluation was conducted, in which all remaining items were approved with a CVI of 1.0, indicating full agreement and eliminating the need for further changes. Authorization was then requested and obtained from the AHRQ team for the exclusion of the aforementioned items, resulting in the adapted HCAHPS version composed of 22 items.

### Response Process

The adapted version was sent to 164 patients, with 33 responses received. Among these, the majority were female (54.5%), aged between 20 and 29 years (30.3%); had up to 12 years of education (33.3%); were hospitalized for two to seven days (66.7%); and were admitted for surgical procedures (n = 14). In the assessment of patients’ comprehension and understanding of the instrument, no participant indicated the need for any modifications.

### Internal Structure

The instrument was distributed to 526 patients, with 304 responses received. Of these, 67.4% were female; 38.5% were between 20 and 29 years old; 37.8% had up to 12 years of education; 73.3% had been hospitalized for two to seven days; and 33.9% were admitted for gynecological or obstetric treatment.

In the exploratory factor analysis, the six-dimension model was initially examined. As shown in Chart 1, the data demonstrated acceptable factorability for model analysis and indicated that the HCAHPS is a unidimensional instrument. Additionally, the explained variance was low (37.9%), suggesting that the six-domain structure of HCAHPS does not adequately represent the latent variable under investigation, as the reference value is above 60%^(9)^.

**Chart 1:** Analysis of sample adequacy measures, explained variance, factor loadings, and communalities of the initial and final models (N = 304), Maringá, Paraná, Brazil, 2021

Sample adequacy measures	Dimensions	Initial Model - six dimensions^*^						One dimension^*^		Final Model - one dimension^*^		
	Matrix determinant	< 0.000001						< 0.000001		0.007673527524456		
	Bartlett’s test of sphericity	853.9 (df = 210; *p* = 0.000010)						853.9 (df = 210; *p* = 0.000010)		382.3 (df = 28; *p* = 0.000010)		
	KMO Coefficient	0.75690 (fair)						0.75690 (fair)		0.80852 (good)		
Explained Variance		37.9711%						38.5340%		70.9059%		
Dimension Indicator		1						1		1		
Final version items		F1	F2	F3	F4	F5	F6	F1	Communality	F1	Communality	
1 - During this hospital stay, how often did the nurses treat you with attention and respect?		-0.767	-0.267	-0.067	0.010	0.108	0.219	-0.766	0.586	0.783	0.613	
2 - During this hospital stay, how often did the nurses listen to you attentively?		-0.885	-0.287	0.047	-0.145	-0.132	0.074	-0.883	0.779	Excluded		
3 - During this hospital stay, how often did the nurses explain things in a way you could understand?		-0.634	-0.452	0.029	-0.057	-0.036	0.070	-0.626	0.392	0.628	0.395	
4 - During this hospital stay, after pressing the call button, how often did you receive help when you needed it?		-0.343	-0.026	-0.379	0.068	-0.121	-0.044	-0.339	0.115	Excluded		
5 - During this hospital stay, how often did the doctors treat you with attention and respect?		-0.688	0.408	0.264	-0.140	-0.106	-0.135	-0.665	0.443	0.603	0.364	
6 - During this hospital stay, how often did the doctors listen to you attentively?		-0.727	0.475	0.329	-0.228	-0.140	0.093	-0.689	0.475	0.636	0.404	
7 - During this hospital stay, how often did the doctors explain things in a way you could understand?		-0.849	0.181	0.238	-0.290	0.100	-0.019	-0.836	0.699	Excluded		
8 - During this hospital stay, how often was your room and bathroom cleaned?		-0.327	0.147	-0.198	0.143	-0.313	0.352	-0.318	0.101	Excluded		
9 - During this hospital stay, how often was the area near your room quiet at night?		-0.491	-0.003	0.022	0.323	-0.318	-0.528	-0.463	0.215	Excluded		
10 - During this hospital stay, did you need help from nurses or other hospital staff to go to the bathroom or use a bedpan?		Item excluded given the absence of results										
11 - How often did you receive help to go to the bathroom or use a bedpan when you needed it?		-0.580	0.160	-0.320	0.096	-0.301	0.212	-0.564	0.318	Excluded		
12 - During this hospital stay, did you receive any medication that you had not taken before?		0.105	0.244	0.016	0.144	-0.169	-0.077	0.106	0.011	Excluded		
13 - Before giving you a new medication, how often did the hospital staff explain the purpose of the medication?		-0.545	-0.103	0.135	0.295	0.213	-0.229	-0.538	0.289	0.557	0.310	
14 - Before giving you a new medication, how often did the hospital staff explain the possible side effects in a way you could understand?		-0.402	0.254	-0.104	0.293	0.292	0.129	-0.393	0.154	Excluded		
15 - After being discharged from the hospital, did you go directly to your home, someone else’s home, or another healthcare facility?		-0.051	-0.074	0.091	0.050	-0.111	0.162	-0.052	0.003	Excluded		
16 - During this hospital stay, did doctors, nurses, or another professional talk to you about whether you would have the help you need after discharge?		0.304	-0.101	-0.018	-0.241	-0.336	-0.074	0.301	0.091	Excluded		
17 - During this hospital stay, did you receive written information about what symptoms or health problems to watch for after discharge?		0.287	-0.209	0.080	-0.133	-0.203	-0.193	0.285	0.081	Excluded		
18 - Using any number from 0 to 10, where 0 is the worst hospital possible and 10 is the best hospital possible, what number would you use to rate this hospital during your stay?		-0.810	-0.185	0.158	0.118	0.047	0.010	-0.816	0.667	0.882		0.777
19 - Would you recommend this hospital to your friends and family?		-0.828	-0.272	0.087	-0.142	0.142	-0.049	-0.828	0.685	0.871		0.758
20 - During this hospital stay, did the staff take into account my preferences and those of my family or caregiver when deciding what my care needs would be after discharge?		-0.694	-0.074	-0.228	0.197	-0.170	-0.088	-0.691	0.478	0.641		0.410
21 - When I was discharged from the hospital, I had a good understanding of my responsibilities for caring for my health?		-0.305	0.081	-0.610	-0.542	0.126	-0.232	-0.277	0.077	Excluded		
22 - When I was discharged from the hospital, I clearly understood the purpose of each of my medications?		-0.458	0.282	-0.298	0.116	0.301	-0.125	-0.444	0.197	Excluded		

Given the indication of one-dimensionality, a one-domain model was tested, showing acceptable factorability for analysis, matrix loadings, and a slight improvement in explained variance (38.5%). However, several inconsistencies were observed in factor loadings and communalities, as presented in Chart 1.

Based on the overall data analysis-including measures of adequacy, dimensionality, factor loadings, and communalities-alongside the content of the HCAHPS instrument, 13 items (59%) out of the 22 (100%) were excluded to identify a more suitable model.

Item exclusions were due to the following inconsistencies: measurement of more than one factor within the same item (items 1, 5, 8, 10, 11, 15 to 17, 19, and 20); lack of consistency in the number of response options (items 4, 10, 12, 15, 16, 17, 18, and 22); and response scales that allowed participants to “skip” to another question (items 10, 1-12, 16, and 17).

It is important to highlight that some items retained in the model also presented wording inconsistencies, such as the presence of two or more factors within the same question (e.g., attention and respect in item 1; friends and family in item 19; and my preferences/my family’s/my caregiver’s in item 20). These items were retained because their exclusion led to model misalignment, negatively impacting sample adequacy measures, explained variance, factor loadings, and communalities of the remaining items, making it impossible to identify a satisfactory model fit.

The inconsistencies in reference values revealed low or negative factor loadings (items 4, 7, 8, 11, 12, 14, 15, 16, 21, and 22) and low communalities (items 4, 8, 11, 12, 14, 15, 16, 17, 21, and 22). Regarding the one-dimensionality testing, the indices for Unidimensional Congruence (0.941), Explained Common Variance (0.807), and Mean of Item Residual Absolute Loadings (0.296) confirmed this assertion.

In Chart 2, it is observed that the Normed Fit Index, Comparative Fit Index, and Root Mean Square Error of Approximation did not yield results (NAn). Meanwhile, McDonald’s Omega exceeded the established reference value, further reinforcing the imprecision of the instrument’s model.

**Chart 2:** Confirmatory factor analysis, reliability indices, quality, and effectiveness (N = 304), Maringá, Paraná, Brazil, 2021

Technique	Applied Indices	Results
Confirmatory Factor Analysis	Normed fit index	Nan^*^
	Comparative Fit Index	-nan(ind)^*^
	Goodness of Fit Index	0.975
	Adjusted Goodness of Fit Index	0.965
	Root Mean Square Error of Approximation	-nan(ind)^*^
Reliability	Standardized Cronbach’s Alpha	0.884224
	McDonald’s Omega	1.019007
	Construct Reliability - Index G H	0.915
Quality and effectiveness	Factor Determinacy Index	0.958
	EAP marginal reliability	0.918
	Sensitivity Ratio	3.355
	Expected percentage of true differences	93.4%

Adapted^(12)^;

* does not present result.

## DISCUSSION

The present study yielded weak results in the evaluation of the internal structure of the HCAHPS, which may be related to poorly worded items and/or response scales, leading to imprecise outcomes. This is because, in addition to influencing participants’ responses, such issues affect the quality of the questionnaire data and the factor structure of the model^(13)^.

To ensure appropriate items and response scales, it is necessary to write them in a simple and objective manner; each item should be aligned with the response scale; and the response options should be presented in ascending order without overlapping^(14)^. These recommendations, however, were not applied in the development of the HCAHPS.

It was also found that nearly half of the items in the instrument measured more than one factor, as evidenced by the use of “and” and/or “or” in the wording of the questions. In addition to this inconsistency, some variables contained more than 20 words, which contradicts established guidelines for instrument content development^(15)^.

Regarding the response scale, it should contain the same number of options to be selected, follow a consistent sequence, and its content should reflect the latent variable^(14)^. These requirements were not considered in the construction of the HCAHPS, as five different response formats were identified throughout the instrument. It is fundamental that the development of instruments, in their entirety, be based on best practices to ensure reliability in the interpretation of results, both by researchers and by users of the instrument^(15)^.

The Brazilian version of the HCAHPS instrument did not converge with the polychoric correlation matrix, which is reported as the most appropriate method for analyzing assessment instruments^(16)^. As a result, it was necessary to use the Pearson correlation matrix, which likely had a negative impact on the internal structure results. One of the prerequisites for using Pearson correlation is that the data be continuous and normally distributed^(17)^, but this principle cannot be met in analyses involving instruments with ordinal and/or nominal data^(15)^.

Regarding studies aimed at validating the HCAHPS in other languages, although the authors claim that the data are valid for their respective countries^(18-20)^, an analysis conducted by the authors of the present study revealed imprecision in the published results. Among other limitations, it was found that the principles of best practices for operationalizing validity evidence were not adequately observed.

Other studies aimed at analyzing the validity of the HCAHPS instrument have also yielded inconsistent results regarding the measurement of patient experience^(18-21)^. In the original HCAHPS pilot study, which used a hypothetical structure of eight domains and 33 items, item-scale correlation analysis revealed no correlation between items and the proposed hypothesis. Several questions showed similar correlations, and there were missing responses, further reinforcing the inconsistency of the test structure^(18)^.

In 2005, a study was published with the objective of identifying parsimonious, reliable, valid, and relevant dimensions of the HCAHPS. Confirmatory factor analysis and internal consistency verification using Cronbach’s Alpha were applied, and several items were excluded for not being related to any factor or to patient experience, among other reasons. After the exclusions, 16 items remained, distributed across seven dimensions. According to the authors of that study, despite the inconsistencies, the reduced model was considered valid and reliable for measuring patient experience in the United States of America^(22)^.

A study conducted using the 33-item version of the instrument applied exploratory factor analysis and found that the use of three dimensions was necessary to achieve parsimonious covariation^(23)^. In 2010, a study aimed at presenting the development, implementation, and dissemination of the HCAHPS, based on 27 items, performed exploratory factor analysis using rincipal components, PROMAX rotation with Kaiser normalization, revealing the existence of six domains^(24)^. However, these techniques contradict best practices for instrument validity as recommended in the literature^(25)^, suggesting that the HCAHPS presents imprecision.

For the present study, the 29-item version available on the official website of the agency responsible for administering the instrument in the United States of America was used. However, the website only provides information related to the psychometric analysis of the pilot study. Therefore, no data regarding the analysis of the latest version were found. In light of the results obtained in this investigation and the studies mentioned, any further discussion of the remaining data presented would be inappropriate due to the inconsistencies identified in the HCAHPS.

### Study limitations

Possible limitations of the study refer to its implementation in a single hospital healthcare service; the sample size being smaller than recommended in the literature; the application of the HCAHPS instrument via social media, whereas the original study recommends distribution by mail; and the absence of psychometricians on the expert committee responsible for content analysis.

It is considered that these limitations did not affect the results obtained, as the qualitative analysis of the instrument’s content revealed issues in item wording and response scale, which were confirmed by the internal structure analysis.

### Contributions to the Field of Nursing

The execution of psychometric studies highlights the importance of the researcher’s commitment to excellence in patient care, guided by valid and reliable instruments. Thus, the knowledge generated by this research may contribute to advancing discussions on sources of validity evidence for instruments, ultimately benefiting nursing care.

## CONCLUSIONS

It is concluded that the HCAHPS instrument is not suitable for use in the Brazilian context due to inconsistencies in item wording and response scale. These inconsistencies may have been exacerbated by the absence of psychometricians on the expert committee responsible for content analysis. The inclusion of professionals with expertise in psychometrics and in the principles outlined by the Standards for Educational and Psychological Testing could have contributed to greater attention to technical aspects during the cross-cultural adaptation process. Therefore, the use of the adapted version of HCAHPS in the present study is not recommended for Brazilian culture.

For future research, the authors suggest the development and validation of instruments that measure patient experience in Brazil, based on best practices and sources of validity evidence as discussed in this study.

## Data Availability

The research data are available in a repository: https://doi.org/10.48331/scielodata.WVILEB.

## References

[1] Garcia IM, Pimentel RRS, Aroni P, Dias AO, Silva LGC, Haddad MCFL. (2022). View of Notifications of incidents related to patient safety in a sentinel university hospital. Ciênc, Cuid Saúde.

[2] Ruggiero AM, Lolatto G. (2021). A jornada da acreditação: série 20 anos.

[3] Báo ACP, Prates CG, Amaral-Rosa MP, Costa DG, Oliveira JLC, Amestoy SC (2023). Patient experience about their safety in the hospital environment. Rev Bras Enferm.

[4] Wolf J, Niederhauser V, Marshburn D, LaVela SL (2014). Defining patient experience. Patient Exp J.

[5] Centers for Medicare & Medicaid Services (2019). Survey Materials - HCAHPS.

[6] Agency for Healthcare Research and Quality (2020). Development of the CAHPS Adult Hospital Survey.

[7] Beaton D, Bombardier C, Guillemin F (2007). Recommendations for the Cross-Cultural Adaptation of the Dash & QuickDash Outcome Measures.

[8] American Psychological Association, National Council on Measurement in Education (2014). Standards for educational and psychological tests.

[9] Alexandre NMC, Coluci MZO. (2011). Content validity in the development and adaptation processes of measurement instruments. Ciên Saúde Coletiva.

[10] Hair JF, Black WC, Babin BJ. (2019). Multivariate data analysis. Cengage Learning Emea;.

[11] Brown TA. (2015). Confirmatory Factor Analysis for Applied Research.

[12] Mialhe FL, Moraes KL, Bado FMR, Brasil VV, Sampaio HADC, Rebustini F. (2021). Psychometric properties of the adapted instrument European Health Literacy Survey Questionnaire short-short form. Rev Latino-Am Enfermagem.

[13] Bulut HC, Bulut O. (2022). Item wording effects in self-report measures and reading achievement: Does removing careless respondents help?. Stud Educ Eval.

[14] Weijters B, Millet K, Cabooter E. (2020). Extremity in horizontal and vertical Likert scale format responses: some evidence on how visual distance between response categories influences extreme responding. Int J Res Market.

[15] Furr RM. (2021). Psychometrics: an introduction. Sage Publications Inc;.

[16] Holgado-Tello FP, Chacón-Moscoso S, Barbero-García I, Vila-Abad E. (2010). Polychoric versus Pearson correlations in exploratory and confirmatory factor analysis of ordinal variables. Qual Quant.

[17] Dalson BFF, Silva S. (2024). Desvendando os Mistérios do Coeficiente de Correlação de Pearson (r). Rev Pol Hoje.

[18] Aoki T, Yamamoto Y, Nakata T. (2020). Translation, adaptation and validation of the Hospital Consumer Assessment of Healthcare Providers and Systems (HCAHPS) for use in Japan: a multicentre cross-sectional study. BMJ Open.

[19] Zun AB, Ibrahim MI, Mokhtar AM, Halim AS, Wan Mansor WNA. (2019). Translation, Cross-Cultural Adaptation, and Validation of the Hospital Consumer Assessment of Healthcare Providers and Systems (HCAHPS) into the Malay Language. Int J Environ Res Public Health, Basel.

[20] Judan-Ruiz EA, Mina RJL, Macindo JRB. (2020). Psychometric Properties of the Filipino Version of Hospital Consumer Assessment of Healthcare Providers and Systems (HCAHPS): a cross-cultural validation study. J Patient Exp.

[21] HCAHPS Three-State Pilot - Study Analysis Results (2003). Guidance Portal.

[22] Keller S, O’Malley AJ, Hays RD, Matthew RA, Zaslavsky AM, Hepner KA, Cleary PD. (2005). Methods Used to Streamline the CAHPS® Hospital Survey. Health Serv Res.

[23] O’Malley AJ, Zaslavsky AM, Hays RD, Hepner KA, Keller S, Cleary PD. (2005). Exploratory Factor Analyses of the CAHPS® Hospital Pilot Survey Responses across and within Medical, Surgical, and Obstetric Services. Health Serv Res.

[24] Giordano LA, Elliott MN, Goldstein E, Lehrman WG, Spencer PA. (2009). Development, Implementation, and Public Reporting of the HCAHPS Survey. Med Care Res Rev.

[25] Osborne JW. (2014). Best practices in exploratory factor analysis. Charleston, Sc. Createspace.

