# Supplementary material for: Validity evidence of the Hospital Consumer Assessment of Healthcare Providers and Systems instrument: Brazilian version
Source: Rev Bras Enferm. 2026 Aug 3;79:e20240526. doi: 10.1590/0034-7167-2024-0526 (PMC13435852; doi:10.1590/0034-7167-2024-0526)
Supplement: Translation

# Translation: Evidências de validade do instrumento *Hospital Consumer Assessment of Healthcare Providers and Systems:* versão brasileira

**DOI:** 10.1590/0034-7167-2024-0526pt

**Authors:** Gislene Aparecida Xavier Reis, Laura Misue Matsuda, Flavio Rebustini, Diovane Ghignatti da Costa, Maria do Carmo Fernandez Lourenço Haddad, Sonia Silva Marcon

**Affiliations:** I Universidade Estadual de Maringá. Maringá, Paraná, Brasil; II Universidade de São Paulo. São Paulo, São Paulo, Brasil; III Universidade Federal de Santa Catarina. Florianópolis, Santa Catarina, Brasil; IV Universidade Estadual de Londrina. Londrina, Paraná, Brasil

**Keywords:** Assistência Centrada no Paciente, Participação do Paciente, Avaliação de Resultados em Cuidados de Saúde, Estudo de Validação, Psicometria.

## Abstract

**Objetivos:**

adaptar e validar o instrumento *Hospital Consumer Assessment of Healthcare Providers and Systems* para a cultura brasileira do Brasil.

**Métodos:**

estudo metodológico que envolveu tradução, adaptação transcultural, validade de conteúdo, processo de resposta e avaliação da estrutura interna de instrumento com seis dimensões. Os dados foram coletados por via remota, com envio de link via *WhatsApp* para acesso ao instrumento adaptado.

**Resultados:**

na adaptação transcultural foi necessária a adequação de alguns termos, e na validade de conteúdo foram excluídos alguns itens e realizadas adequações gramaticais. No processo de resposta não houve indicação de alterações, na análise fatorial exploratória excluíram-se 13 (59%) itens, e foram observados indícios de que o instrumento possuía problemas em sua estrutura e era composto por uma única dimensão. Na análise fatorial confirmatória, ratificou-se que o instrumento é unidimensional e impreciso.

**Conclusões:**

não é recomendado o uso desta versão adaptada para a cultura brasileira.

## INTRODUÇÃO

Com o advento da ampla disseminação de informações, no que se refere ao sistema de saúde, os pacientes se apresentam cada vez mais conhecedores dos seus direitos. Com isso, é primordial que os serviços dessa área realizem iniciativas voltadas à qualidade e à segurança do paciente^(1,2)^.

Dentre as estratégias para avaliar a qualidade do cuidado, destacam-se aquelas em que o paciente relata sua experiência acerca da assistência recebida^(2,3)^. A saber, a experiência do paciente (EP) compreende o resultado das suas interações com o serviço de saúde e com a cultura organizacional durante a sua jornada de cuidado^(4)^.

Outra forma de avaliar é mediante a aplicação de instrumentos que abordam especificamente a experiência do paciente. E um deles é o *Hospital Consumer Assessment of Healthcare Providers and Systems* (HCAHPS^®^), que foi elaborado e validado em 2008 pela *Agency for Healthcare Research and Quality* (AHRQ), em parceria com *Centers for Medicare & Medicaid Services* (CMS)^(5)^ e *Agency For Healthcare Research and Quality* ^(6)^. Além de avaliar a EP, o HCAHPS^®^ possibilita que as organizações hospitalares recebam subsídios financeiros do governo americano, que os usuários dos serviços de saúde escolham o hospital onde desejam receber assistência médica, e que haja comparação do desempenho entre os hospitais e aumento da transparência da qualidade do cuidado entre os serviços de saúde^(5,6)^.

Torna-se oportuno validar tal instrumento para o idioma português brasileiro, não só pela importância da avaliação da EP para a qualidade e segurança no cuidado, mas também porque os resultados podem ser úteis para o hospital, visto que o questionário aborda aspectos abrangentes acerca da assistência recebida, além do fato de ser autoaplicável.

O presente estudo se pauta na questão: O instrumento *Hospital Consumer Assessment of Healthcare Providers and Systems*, submetido ao processo de adaptação transcultural e de validade, apresenta evidências de precisão para o idioma português do Brasil?

## OBJETIVOS

Validar o instrumento *Hospital Consumer Assessment of Healthcare Providers and Systems* para o idioma português do Brasil.

## MÉTODOS

### Aspectos éticos

Todos os preceitos éticos que envolvem pesquisas com seres humanos foram respeitados e a proposta desta investigação foi aprovada pelo comitê de ética da instituição signatária (Parecer nº 4.608.997).

### Cenário de estudo e período

Hospital de alta complexidade, situado na região Sul do Brasil, de caráter filantrópico, com 311 leitos, dos quais 60% são destinados a pacientes do Sistema Único de Saúde. Realiza atendimentos nas unidades de clínica cirúrgica, pediatria, ginecologia e obstetrícia, terapia intensiva adulta, pediátrica e neonatal, e possui selo de qualidade nível II, emitido pela Organização Nacional de Acreditação. Os dados foram coletados entre os meses de abril e outubro de 2021.

### Tipo de estudo

Estudo metodológico que envolveu adaptação transcultural^(7)^ e validação das propriedades psicométricas^(8)^ do HCAHPS, cuja versão original do instrumento é constituída por 29 questões distribuídas em seis domínios/dimensões: comunicação com a equipe de enfermagem (04); comunicação com a equipe médica (03); ambiente hospitalar (02); experiência no hospital (05); aspectos da alta hospitalar (06); avaliação geral do hospital (02); caracterização sociodemográfica e estado de saúde atual do paciente (07). Esta pesquisa foi estruturada de acordo com o *Revised Standards for Quality Improvement Reporting Excellence* (SQUIRE).

### Adaptação Transcultural do *Hospital Consumer Assessment of Healthcare Providers and Systems*

O HCAHPS foi traduzido com autorização de seus autores e adaptado transculturalmente (ATC)^(7)^, seguindo as etapas de tradução, síntese das traduções, retrotradução, avaliação por Comitê de Especialistas, pré-teste da versão adaptada e validação final pelos autores originais. Na tradução, dois tradutores bilíngues brasileiros realizaram traduções independentes, sendo um especializado na área e outro “ingênuo”, sem conhecimento prévio dos conceitos, e ambos emitiram relatórios referentes a esse processo. A partir dessas traduções, a pesquisadora, com apoio dos tradutores, elaborou uma versão consenso.

Na retrotradução, dois tradutores nativos de língua inglesa, desconectados do tema, traduziram novamente o instrumento e produziram relatórios. O Comitê de especialistas^(9)^, composto por 11 membros (incluindo tradutores, professor de letras e especialistas em segurança do paciente), foi selecionado por meio do currículo Lattes, site da REBRAENSP e LinkedIn, com base em titulação (mestrado ou doutorado) e experiência mínima de um ano na área. Após aceite, os participantes receberam, de forma online, o Termo de Consentimento e documentos relativos ao processo de adaptação, e responderam a um questionário sobre equivalência idiomática, semântica, experimental e conceitual.

### Validade de conteúdo

Todo o material produzido durante a adaptação transcultural foi enviado para análise de 11 especialistas, sendo: um tradutor, um professor de letras e nove especialistas em segurança do paciente, experiência ou cuidado centrado no paciente, com pelo menos seis meses de atuação prática ou acadêmica. O Comitê não incluiu psicometristas, cuja contribuição poderia fortalecer a análise técnica e estatística do instrumento. Essa ausência ocorreu porque, na época, a pesquisadora ainda não tinha domínio completo dos critérios metodológicos recomendados pelo *Standards for Educational and Psychological Testing* ^(8)^, que destacam a importância da participação de psicometristas nesses processos.

Após a análise qualitativa, realizou-se a análise quantitativa por meio do Índice de Validade de Conteúdo (IVC-i) de cada item, calculado pela fórmula: IVC-i = número de respostas “3” ou “4” / número total de respostas. Foi considerado aceitável o escore mínimo de 0,80, preferencialmente acima de 0,90. As sugestões dos especialistas foram incorporadas, resultando na versão pré-final do HCAHPS, aprovada pela equipe da AHRQ.

### Processo de resposta

A versão pré-final do HCAHPS foi enviada, via WhatsApp, a pacientes que atendiam aos critérios do CMS^(5)^ e AHRQ^(6)^: idade ≥ 18 anos, internação clínica, cirúrgica ou obstétrica e alta entre 48 horas e seis semanas. Não foram incluídos pacientes sem contato por WhatsApp ou que não responderam a pelo menos três tentativas de contato. A identificação de possíveis participantes ocorreu por meio de análise diária de prontuários eletrônicos, considerando diagnóstico, contato telefônico com WhatsApp e data da alta. Os pacientes foram convidados a participarem do estudo por mensagem escrita no WhatsApp que informava sobre o objetivo, forma de coleta dos dados e tempo estimado para responder ao questionário.

Aqueles que concordaram em participar receberam, pelo mesmo canal, o link com o Termo de Consentimento, Questionário Sociodemográfico, Instruções, e a versão pré-final do HCAHPS. Após o preenchimento, foram realizadas entrevistas telefônicas com um questionário estruturado para avaliar a compreensão dos itens. Os dados foram registrados em planilha do *Microsoft Excel* ^®^ e analisados por meio de frequência absoluta e relativa. Todos os participantes demonstraram compreensão dos itens do instrumento, concluindo-se assim a etapa de pré-teste.

### Estrutura interna

Para avaliar a estrutura interna, a versão final do instrumento foi aplicada em uma nova amostra de pacientes que atendiam aos mesmos critérios de inclusão estabelecidos previamente. Os dados foram compilados em planilha do *Microsoft Excel* ^®^, exportados para o *software Factor Analysis* (versão 10.10.03) e submetidos à análise fatorial exploratória (AFE), seguida da análise fatorial confirmatória (AFC).

Inicialmente, foi realizada a correlação de Pearson, já que os dados não convergiram na matriz policórica devido às seguintes inconsistências nas escalas de resposta: itens com respostas dicotômicas (10, 12, 16, 17); escala Likert de cinco pontos (4 e 22); itens com opção de “saltar” (11, 13, 14, 16, 17); item com três categorias (15); e item com 11 categorias (18). Além disso, os itens com opção de “saltar” geraram 72,7% de dados ausentes, aumentando a inconsistência dos resultados. Vale destacar que a análise inicial só foi possível após a exclusão do item 10, pois o *software* não processava os cálculos com sua presença.

Foram utilizados os seguintes critérios para verificação da adequação da amostra: coeficiente *Kaiser-Meyer-Olkin* (KMO) > 0,70; teste de esfericidade de Bartlett com p < 0,05; determinante da matriz > 0,00001 e bootstrapping com 5.000 reamostragens^(10)^.

Utilizou-se a *Parallel Analysis* na análise fatorial exploratória a fim de examinar a dimensionalidade do HCAHPS, *Unweighted Least Squares* (ULS) para técnica de extração, rotação fatorial Promin para testar a multidimensionalidade e nenhuma técnica de rotação para unidimensionalidade^(10)^. Os fatores foram avaliados quanto à carga fatorial com valores entre 0.30 a 0.70, comunalidade 0.40 a 0.70 e, *Heywood case* < 0% ou > 100%. No que tange à AFC, considerou-se: valor próximo de 1 para *Normed fit index* e *Comparative Fit Index*, para o *Goodness of Fit Index* e *Adjusted Goodness of Fit Index* valor > 0.90 e, para *Root Mean Square Error of Approximation*, valores próximos de zero^(11)^.

A confiabilidade foi analisada por meio do *Standardized Cronbach’s Alpha* e *McDonald’s Ômega*, com índices entre 0.70 a 0.90 e *Construct Reliability - Index G H* (Latente e observada), com valores > 0.80^(11)^. Para confirmar a dimensionalidade do HCAHPS, foram estabelecidas as técnicas de *Unidimensional Congruence* ≥ 0.95, Explained Common Variance ≥0.85, e Mean of item residual absolute loading ≤0.30, respectivamente^(11)^.

Quanto à qualidade e à efetividade do modelo, consideraram-se *Factor Determinacy Index* > 0.90, *Confiabilidade Marginal EAP* > 0.80, *Sensivity Ratio* > 2, e *Expected percentage of true differences* > 90%^(10)^. Cabe citar que tais sequências de técnicas foram guiadas pelo estudo de Mialhe et al.^(12)^, cujos valores de referência da AFE se ancoraram em Hair et al.^(10)^ e Brown^(11)^ para AFC.

## RESULTADOS

### Adaptação transcultural

Durante reunião para consenso das traduções, estabeleceu-se a utilização dos termos “pressionei” ao invés de “acionei; “próxima” e não “ao redor”; “comadre” e não “urinol”. A versão produzida do HCAHPS na etapa de adaptação transcultural está disponível no QRcode na Figura 1.

**Figura 1:** Versão produzida do *Hospital Consumer Assessment of Healthcare Providers and Systems* na etapa de adaptação transcultural, Maringá, Paraná, Brasil, 2021

### Validade de Conteúdo

Dos 11 especialistas/juízes, a maioria era do sexo feminino (81,8%), enfermeira (72,7%), tinha experiência em qualidade, segurança e/ou experiência do paciente no ambiente hospitalar (90,9%), mais da metade tinha o título de doutor (54,5%) e residia na região sul do país (54,5%). Na primeira rodada de avaliação, o Comitê de Especialistas julgou a necessidade de ajustes gramaticais nos itens 2, 11, 16 e 20, para atender aos critérios de equivalência semântica, experimental, idiomática e conceitual. Além disso, os itens 23 a 29 foram considerados incoerentes e sem relação com a variável latente, sendo assim excluídos. O IVC obtido foi de 0,40.

Após as adequações propostas, foi realizada uma segunda rodada de avaliação, na qual todos os itens restantes foram aprovados com IVC de 1,0, indicando concordância plena e dispensando novas alterações. Assim, foi solicitada e obtida autorização da equipe da AHRQ para a exclusão dos referidos itens, resultando na versão adaptada HCAHPS, composta por 22 itens.

### Processo de resposta

A versão adaptada foi encaminhada a 164 pacientes, obtendo-se retorno de 33. Destes, a maioria era do sexo feminino (54,5%), com idade entre 20 a 29 anos (30,3%), com até 12 anos de estudo (33,3%), tempo de internação entre dois e sete dias (66,7%), e internados para procedimento cirúrgico (n= 14). Na averiguação sobre a compreensão e entendimento dos pacientes sobre o instrumento, nenhum participante indicou necessidade de realizar alterações.

### Estrutura interna

O instrumento foi encaminhado a 526 pacientes, com retorno de 304. Destes, 67,4% eram do sexo feminino, 38,5% tinham idade entre 20 a 29 anos, 37,8% com até 12 anos de estudo, 73,3% permaneceram internados entre dois e sete dias, sendo 33,9% para tratamento ginecológico ou obstétrico.

Na análise fatorial exploratória, inicialmente investigou-se o modelo de seis dimensões. Conforme consta no Quadro 1, verificou-se justa fatorabilidade para análise do modelo, e indicação de que o HCAHPS é um instrumento unidimensional. Além disso, a variância explicada foi baixa (37,9%), o que indica que o HCAHPS, com seis domínios, não representa de modo profundo a variável latente investigada pois o valor de referência é superior a 60%^(9)^.

**Quadro 1:** Análise das medidas de adequação da amostra, variância explicada, cargas fatoriais e comunalidades do modelo inicial e final (N=304), Maringá, Paraná, Brasil, 2021

Medidas de adequação da amostra	Dimensões	Modelo inicial - seis dimensões^*^						Uma dimensão^*^		Modelo final - uma dimensão^*^		
	Determinante matriz	< 0,000001						< 0,000001		0,007673527524456		
	Esfericidade de Bartlett	853,9 (df = 210; *p* = 0,000010)						853,9 (df = 210; *p* = 0,000010)		382,3 (df = 28; *p* = 0,000010)		
	Índice KMO	0,75690 (justo)						0,75690 (justo)		0,80852 (bom)		
Variância explicada		37,9711%						38,5340%		70,9059%		
Indicação de dimensão		1						1		1		
Itens da versão final		F1	F2	F3	F4	F5	F6	F1	Comunalidade	F1	Comunalidade	
1- Nesta internação, com que frequência as enfermeiras o(a) trataram com atenção e respeito?		-0,767	-0,267	-0,067	0,010	0,108	0,219	-0,766	0,586	0,783	0,613	
2- Nesta internação, com que frequência os enfermeiros ouviram você com atenção?		-0,885	-0,287	0,047	-0,145	-0.132	0,074	-0,883	0,779	Excluída		
3- Nesta internação, com que frequência os enfermeiros explicaram as coisas de uma forma que você pudesse compreender?		-0,634	-0,452	0,029	-0,057	-0.036	0,070	-0,626	0,392	0,628	0,395	
4- Nesta internação, depois de pressionar a campainha de chamada, com que frequência você recebeu ajuda no momento que solicitou?		-0,343	-0,026	-0,379	0,068	-0.121	-0,044	-0,339	0,115	Excluída		
5- Nesta internação, com que frequência os médicos o(a) trataram com atenção e respeito?		-0,688	0,408	0,264	-0,140	-0.106	-0,135	-0,665	0,443	0,603	0,364	
6- Nesta internação, com que frequência os médicos ouviram você com atenção?		-0,727	0,475	0,329	-0,228	-0.140	0,093	-0,689	0,475	0,636	0,404	
7- Nesta internação, com que frequência os médicos explicaram as coisas de forma que você pudesse compreender?		-0,849	0,181	0,238	-0,290	0.100	-0,019	-0,836	0,699	Excluída		
8-Nesta internação, com que frequência foi realizada a limpeza do seu quarto e do banheiro?		-0,327	0,147	-0,198	0,143	-0.313	0,352	-0,318	0,101	Excluída		
9-Nesta internação, com que frequência a área próxima ao seu quarto permaneceu em silêncio à noite?		-0,491	-0,003	0,022	0,323	-0.318	-0,528	-0,463	0,215	Excluída		
10-Nesta internação, você precisou da ajuda de enfermeiras ou outra equipe do hospital para ir ao banheiro ou usar uma comadre?		Item excluído devido à ausência de resultados										
11-Com que frequência você recebeu ajuda para ir ao banheiro ou usar uma comadre quando precisou?		-0,580	0,160	-0,320	0,096	-0,301	0,212	-0,564	0,318	Excluída		
12-Nesta internação, você recebeu algum medicamento que não havia tomado antes?		0,105	0,244	0,016	0,144	-0,169	-0,077	0,106	0,011	Excluída		
13-Antes de lhe dar um novo medicamento, com que frequência a equipe do hospital informou a finalidade do medicamento?		-0,545	-0,103	0,135	0,295	0,213	-0,229	-0,538	0,289	0,557	0,310	
14-Antes de lhe dar um novo medicamento, com que frequência a equipe do hospital informou os possíveis efeitos colaterais de forma que você pudesse compreender?		-0,402	0,254	-0,104	0,293	0,292	0,129	-0,393	0,154	Excluída		
15-Depois de ter alta do hospital, você foi diretamente para sua casa, para a casa de outra pessoa ou para outra unidade de saúde?		-0,051	-0,074	0,091	0,050	-0,111	0,162	-0,052	0,003	Excluída		
16-Nesta internação, médicos, enfermeiros ou outro profissional conversou com você para saber se teria a ajuda que precisa após ter recebido alta hospitalar?		0,304	-0,101	-0,018	-0,241	-0,336	-0,074	0,301	0,091	Excluída		
17-Durante esta internação no hospital, você recebeu informações por escrito sobre quais sintomas ou problemas de saúde deveria observar após ter recebido alta hospitalar?		0,287	-0,209	0,080	-0,133	-0,203	-0,193	0,285	0,081	Excluída		
18-Usando qualquer número de 0 a 10, onde 0 é o pior hospital possível e 10 é o melhor hospital possível, qual número você usaria para avaliar este hospital durante sua internação?		-0,810	-0,185	0,158	0,118	0,047	0,010	-0,816	0,667	0,882		0,777
19-Você recomendaria este hospital aos seus amigos e familiares?		-0,828	-0,272	0,087	-0,142	0,142	-0,049	-0,828	0,685	0,871		0,758
20-Nesta internação, a equipe levou em consideração minhas preferências e as de minha família ou cuidador ao decidir quais seriam minhas necessidades de cuidados após receber alta?		-0,694	-0,074	-0,228	0,197	-0,170	-0,088	-0,691	0,478	0,641		0,410
21-Quando recebi alta hospitalar, tive uma boa compreensão das minhas responsabilidades para O CUIDADO da minha saúde?		-0,305	0,081	-0,610	-0,542	0,126	-0,232	-0,277	0,077	Excluída		
22-Quando recebi alta hospitalar, entendi claramente a função de cada um dos meus medicamentos?		-0,458	0,282	-0,298	0,116	0,301	-0,125	-0,444	0,197	Excluída		

Diante da indicação de unidimensionalidade, realizou-se testagem para um domínio, identificando-se justa fatorabilidade para análise do modelo, carregamento da matriz fatorial, discreta melhora na variância explicada (38,5%) dos itens e diversas inconsistências quanto às cargas fatoriais e comunalidades, conforme apresentado no Quadro 1.

Pela análise do conjunto dos dados, composta pelas medidas de adequação, dimensionalidade, cargas fatoriais e comunalidade, atrelado ao conteúdo do instrumento HCAHPS, 13 (59%) dos 22 (100%) itens foram excluídos, para identificar o modelo adequado.

As exclusões dos itens ocorreram pelas seguintes inconsistências: mensuração de mais de um fator no mesmo item (itens 1, 5, 8, 10, 11, 15 ao 17, 19 e, 20), ausência de padrão na quantidade de opções de respostas (itens 4, 10, 12, 15, 16, 17, 18 e 22) e escala de resposta com a possibilidade de “saltar” para outra questão (itens 10, 1-12; 16 e 17).

Ressalta-se que alguns dos itens que permaneceram no modelo também apresentaram inconsistências na redação como por exemplo presença de dois ou mais fatores em uma mesma questão (atenção e respeito no item 1, amigos e familiares no item 19, e minhas preferências/de minha família/ou meu cuidador no item 20). Esses itens permaneceram porque, ao realizar suas exclusões, o modelo do instrumento apresentava desajustes com impactos negativos nas medidas de adequação da amostra, variância explicada, cargas fatoriais e comunalidades dos outros itens, não sendo possível identificar ajuste satisfatório do instrumento.

As incongruências nos valores de referências demonstraram cargas fatoriais baixas ou negativas (itens 4, 7, 8, 11, 12, 14, 15, 16, 21 e 22) e baixa comunalidade (itens 4, 8, 11, 12, 14, 15, 16, 17, 21 e 22). Já no que diz respeito à testagem da unidimensionalidade, identificou-se que os índices de Congruência Unidimensional (0.941), Variância Comum Explicada (0.807) e Cargas Absolutas Residuais (0.296) ratificam essa afirmação.

No Quadro 2, observa-se que os índices *Normed fit index, Comparative Fit Index* e *Root Mean Square Error of Approximation* não apresentaram resultados (NAn). Já o *McDonald’s Ômega* excedeu o valor estipulado de referência, o que reforça a imprecisão no modelo do instrumento.

**Quadro 2:** Análise fatorial confirmatória, índices de confiabilidade, qualidade e efetividade (N=304), Maringá, Paraná, Brasil, 2021

Técnicas	Índices aplicados	Resultados
Análise fatorial confirmatória	*Normed fit index*	Nan^*^
	*Comparative Fit Index*	-nan(ind)^*^
	*Goodness of Fit Index*	0,975
	*Adjusted Goodness of Fit Index*	0,965
	*Root Mean Square Error of Approximation*	-nan(ind)^*^
Confiabilidade	*Standardized Cronbach’s Alpha*	0,884224
	*McDonald’s Ômega*	1,019007
	*Construct Reliability - Index G H*	0,915
Qualidade e efetividade	*Factor Determinacy Index*	0,958
	*EAP marginal reliability*	0,918
	*Sensivity Ratio*	3,355
	*Expected percentage of true diferences*	93,4%

Adaptado^(12)^;

* não apresenta resultados.

## DISCUSSÃO

O presente estudo obteve resultados frágeis na avaliação da estrutura interna do HCAHPS, o que pode estar relacionado com a redação inadequada dos itens e/ou da escala de resposta, desencadeando resultados imprecisos. Isso porque, além de influenciar as respostas dos participantes, afeta a qualidade dos dados do questionário e a estrutura fatorial do modelo^(13)^.

Para proporcionar itens e escala de respostas adequadas, faz-se necessário redigi-los de modo simples e objetivo. Além disso, o item deve possuir relação com a escala de respostas e as opções de respostas devem ser apresentadas em ordem crescente e sem sobreposição^(14)^. Estas recomendações, portanto, não foram aplicadas na elaboração do HCAHPS.

Constatou-se também que quase a metade dos itens do referido instrumento mensurava mais de um fator nas variáveis, e isto foi evidenciado pelo uso do “e” e/ou “ou” no enunciado das questões. Além da referida inconsistência, identificou-se que algumas variáveis eram compostas por mais de 20 palavras, e isto se contrapõe às orientações sobre elaboração de conteúdo de instrumentos^(15)^.

Quanto à escala de resposta, esta deve possuir o mesmo número de opções para serem assinaladas, mesma sequência, e o conteúdo deve refletir a variável latente^(14)^. Estes requisitos não foram considerados na construção do HCAHPS porque foi constatada a presença de cinco diferentes opções de resposta ao longo do instrumento. É basilar que a elaboração de instrumentos, em sua totalidade, seja fundamentada em boas práticas de modo a proporcionar fidedignidade na interpretação dos resultados, tanto por pesquisadores como pelos usuários do instrumento^(15)^.

O instrumento HCAHPS, versão brasileira, não convergiu para a matriz de correlação policórica, que é reportada como a mais indicada para a análise de instrumentos de avaliação^(16)^. Com isso, foi necessário utilizar a matriz de correlação de Pearson que, certamente, impactou de modo negativo nos resultados da estrutura interna. Uma das premissas para a utilização da referida correlação é que os dados sejam contínuos e tenham distribuição normal^(17)^, mas este princípio não é possível de ser cumprido nas análises de instrumentos em que os dados são ordinais e/ou nominais^(15)^.

Com relação a estudos com objetivo de validar o HCAHPS para outros idiomas, apesar de os autores afirmarem que os dados são válidos para os respectivos países^(18-20)^, em análise realizada pelos autores do presente estudo constatou-se imprecisão nos resultados dos estudos publicados. Isso porque, dentre outras limitações, identificou-se a não observância quanto aos princípios das boas práticas para operacionalização das evidências de validade.

Outras pesquisas com intuito de analisar a validade do instrumento HCAHPS também obtiveram resultados inconsistentes para a mensuração da experiência do paciente^(18-21)^. No estudo piloto do HCAHPS original, em que foi utilizada estrutura hipotética, de oito domínios e 33 itens por meio de correlação item-escala, verificou-se que não houve correlação entre o item e a hipótese, diversas questões tiveram correlações semelhantes, e houve ausências de respostas, o que reforçou a inconsistência da estrutura do teste^(18)^.

Em 2005, foi publicado um estudo com o objetivo de identificar dimensões parcimoniosas, confiáveis, válidas e relevantes do HCAHPS. Para isso aplicou-se a análise fatorial confirmatória e a verificação da consistência interna, por meio de *Alpha de Cronbach*, sendo excluídos diversos itens por não se relacionarem com nenhum fator, não terem relação com a experiência do paciente, dentre outros motivos. Após as exclusões, restaram 16 itens distribuídos em sete dimensões. De acordo com os autores do referido estudo, apesar das inconsistências, o modelo reduzido foi considerado válido e confiável para mensurar a experiência do paciente nos Estados Unidos da América^(22)^.

Em uma pesquisa realizada com o instrumento de 33 itens em que foi aplicada a análise fatorial exploratória, os autores constataram que a utilização de três dimensões era necessária para se obter covariação parcimoniosa^(23)^. Em 2010, uma pesquisa com intuito de apresentar o desenvolvimento, implementação e divulgação do HCAHPS, pautada em 27 itens, realizou a análise fatorial exploratória com componentes principais, rotação PROMAX com normalização de Kaiser, evidenciando-se a existência de seis domínios^(24)^. Contudo, essas técnicas contrapõem as boas práticas para a validade de instrumentos recomendados na literatura^(25)^, e isso sugere que o HCAHPS se apresenta impreciso.

Para a realização do presente estudo, utilizou-se a versão com 29 itens, disponibilizada no site oficial do órgão responsável pela administração do instrumento nos Estados Unidos da América. Contudo, neste site somente são disponibilizadas informações referentes à análise psicométrica do estudo piloto. Portanto, informações referentes à análise da última versão não foram encontradas. Diante dos resultados obtidos nesta investigação, e das pesquisas mencionadas, quaisquer discussões acerca dos demais dados apresentados seriam inadequadas em razão das inconsistências constatadas no HCAHPS.

### Limitações do estudo

Possíveis limitações do estudo referem-se à sua operacionalização em um único serviço de saúde hospitalar, ao tamanho amostral inferior ao recomendado pela literatura, à aplicação do instrumento HCAHPS por meio de mídia social, visto que o estudo original indica o envio do instrumento por meio de correspondência, e à ausência de psicometristas no Comitê de Especialistas responsável pela análise de conteúdo.

Considera-se que as limitações referidas não alteram os resultados encontrados, pois por meio da análise qualitativa do conteúdo do instrumento constatou-se a presença de problemas na redação dos itens e na escala de resposta, o que foi ratificado pela análise da estrutura interna.

### Contribuições para a área da enfermagem

A realização de estudos psicométricos suscita a importância do comprometimento do pesquisador quanto à diligência pela excelência na qualidade da assistência ao paciente, norteada por instrumentos válidos e precisos. Assim, o conhecimento gerado por essa pesquisa pode contribuir para o avanço das discussões sobre as fontes de evidências de validade de instrumentos, beneficiando a assistência de enfermagem.

## CONCLUSÕES

Conclui-se que o instrumento HCAHPS não se apresenta preciso para uso no contexto brasileiro porque possui inconsistências na redação dos itens e na escala de respostas. Essas inconsistências podem ter sido agravadas pela ausência de psicometristas no Comitê de Especialistas responsável pela análise de conteúdo. A inclusão de profissionais com expertise em psicometria e nos pressupostos do *Standards for Educational and Psychological Testing* poderia ter contribuído para uma maior atenção aos aspectos técnicos durante o processo de adaptação transcultural. Diante disso, não se recomenda a utilização da versão adaptada do HCAHPS no presente estudo para a cultura brasileira.

Para futuras investigações, os autores sugerem o desenvolvimento e a validação de instrumentos que mensurem a experiência de pacientes no Brasil fundamentados nas boas práticas das fontes de evidências de validade abordadas neste estudo.

## Data Availability

Os dados de pesquisa estão disponíveis em repositório: https://doi.org/10.48331/scielodata.WVILEB.
